# Progress in Surgery

**Published:** 1898-07-23

**Authors:** 


### PROGRESS IN SURGERY.

### GENITO-URINARY SURGERY.

**Historical.**—The history of the development of renal surgery has recently been traversed by Mr. Henry Morris, in his Hunterian Lectures,[1] to which the reader may refer for a very ample exposition. Considering the present state of renal surgery, one marvels at the fact that 20 years ago its infancy had scarcely dawned. Nephropexy rapidly succeeded nephrectomy and nephrolithotomy, and within the last few years partial excision of a portion of the kidney has become established, the renal tissue healing well, and the tendency to urinary fistula proving to be slight. Moreover, the ureter is now successfully treated by operation, calculi being removed, strictures cured, and kinks corrected. For nephropexy Mr. Morris has discarded all other forms of suture in favour of silk, and he speaks highly of the permanency of results therewith. Excisions of portion of the kidney have been performed for tuberculous abscesses, for localised miliary tubercles, and for partial laceration, the typical operation being the excision of a wedge-shaped piece. Fenger, of Chicago, performed the first successful ureteroplastic operation for kink in 1892, and soon performed a similar operation for stricture. Attention may be drawn to the Heineke-Mikulicz suture for the relief of strictured hollow viscera figured in the reference.[1]

**Moveable Kidney.**—From an experience of 21 cases, some lost sight of, Dr. Symons Eccles advises the rest cure combined with massage, before resorting to operation, in order to give tone and to further the redeposition of supporting fat. He finds the condition commonest in those wasted and worn by anxiety.[2] Two new operations have been lately devised, one by Reed, of Columbus, who opens the abdomen from the front and transfixes the kidney to the posterior abdominal wall, tying the sutures over a gauze pad on the skin. The other operation is that of Senn, of Chicago, which has the advantage of being sutureless. It consists of a posterior incision down to the kidney, scarification of its surface, removal of part of the fatty capsule, and support of the lower end of the kidney by an iodoform gauze plug, the ends of which are tied over another plug placed in the wound. The cavity is allowed to granulate. Mr. Bidwell has used Vulliett's method in three cases, and speaks well of the operation, which consists of fixation of the kidney by a suture obtained from the patient's own latissimus dorsi. Dr. Robert Weir, New York, holds that there are two conditions for success—(*a*) a suture material which will maintain its integrity a considerable time; (*b*) allowing at least a portion of the wound to heal by granulation.

**Limits of Nephrectomy.**—The life of the patient depends largely upon the adequacy of the other kidney, which may fail or become diseased, even though apparently healthy.[3] The presence and condition of the other kidney are considered by Dr. Geo. Edebohls, of New York.[4] He cites a case where there was an enlarged gall-bladder but no kidney on the right side. Cystoscopy of the bladder, though useful, may not suffice; the ureteral orifice may not be seen; the pus from a kidney may only be discharged intermittently, and hematuria of constitutional origin may occur from the kidneys alternately. Catheterisation of the ureters may be difficult, and "has its shortcomings, drawbacks, and contraindications, which unfortunately greatly impair the usefulness and efficiency of what would otherwise be a nearly ideal method of obtaining information." In tuberculosis and in pyuria from any cause this method is absolutely contraindicated as dangerous. Skiagraphy is proving of valuable assistance in diagnosis. It is now proposed that before performing nephrectomy the other kidney shall be explored thoroughly, say through the loin. Edebohls places the patient prone on a kidney cushion, and incises parallel to and just external to the erector spinæ. Exploration of the other kidney by this means is the surest means of determining its presence and condition, and its risks are scarcely greater than catheterization of the ureters.

A successful case of nephrectomy for ruptured kidney is recorded by Mr. Moynihan, of Leeds, who finds that primary nephrectomy reduces the mortality from over 40 per cent. to 20 per cent.[5]

**Renal Calculus.**—Early conservative operations are now much more frequent, with corresponding diminished mortality. Henry Morris finds that whereas the mortality of nephrolithotomy is only 3 per cent., or less, that of nephrectomy for calculus is 30 per cent.[6] Even if no calculus be found on exploring the kidney for symptoms referable to the kidney, some other condition may be found, and the patient thus often derives benefit or cure. As instance, adhesions, mobility, cysts, hæmorrhages, abscesses, &c., all of which may give rise to similar signs. Extra-renal conditions must be excluded, *e.g.*, spinal disease, aneurism, gall-stones, visceral adhesions.

Lurking calculi may exist unsuspected, and create insidious renal havoc. They may grow to such a size as to be felt per abdomen, and yet be painless. Mr. Morris is not a believer in the pain being referred to the opposite kidney. Indeed, he regards pain as the best indication as to the side for operation. Unsuspected calculi may cause symptoms eflected to other organs, *e.g.*, the ovaries, uterus, bladder, to anomalous fever and prostration, whilst they progressively destroy the renal parenchyma.

The Röntgen rays have proved of some use in diagnosis. Mr. Fripp was unable to find a stone weighing half an ounce upon exploration of a kidney, but a week later he found it by the aid of skiagraphy.

When nephrolithotomy is delayed so that calculous pyonephrosis results, fistulæ are very apt to follow operation. Moreover, very small calculi, besides causing severe hæmorrhage, may perniciously affect the kidney.

[1] Brit. Med. Jour., Mar. 26, 1898. [2] Lancet, Jan. 29, 1898. [3] Med. Rec., April 23, 1898. [4] Medical Soc. of New York, Jan. 26, 1898. [5] Brit. Med. Assn. Yorkshire Branch, Jan. 26, 1898. [6] Brit. Med. Jour., April 9, 1898.

